# Frequent induction of chromosomal aberrations in *in vivo* skin fibroblasts after allogeneic stem cell transplantation: hints to chromosomal instability after irradiation

**DOI:** 10.1186/s13014-015-0576-4

**Published:** 2015-12-30

**Authors:** G. Massenkeil, P. Zschieschang, G. Thiel, P. G. Hemmati, V. Budach, B. Dörken, J. Pross, R. Arnold

**Affiliations:** Department of Hematology, Oncology and Tumor Immunology, Charité Universitätsmedizin Berlin, Berlin, Germany; Institute for Medical Genetics, Charité Universitätsmedizin Berlin, Berlin, Germany; Clinic for Radiation Oncology, Charité Universitätsmedizin Berlin, Berlin, Germany; Present address: Department of Internal Medicine, Clinic for Hematology and Oncology, Klinikum Guetersloh, Guetersloh, Germany; Present address: Medical practice for Human Genetics, Friedrichstrasse, Berlin, Germany

**Keywords:** Chromosomal aberrations, Skin fibroblasts, Allogeneic stem cell transplantation, Total body irradiation, Radiation-induced chromosomal instability, Secondary malignancies

## Abstract

**Background:**

Total body irradiation (TBI) has been part of standard conditioning regimens before allogeneic stem cell transplantation for many years. Its effect on normal tissue in these patients has not been studied extensively.

**Method:**

We studied the *in vivo* cytogenetic effects of TBI and high-dose chemotherapy on skin fibroblasts from 35 allogeneic stem cell transplantation (SCT) patients. Biopsies were obtained prospectively (*n* = 18 patients) before, 3 and 12 months after allogeneic SCT and retrospectively (*n* = 17 patients) 23–65 months after SCT for G-banded chromosome analysis.

**Results:**

Chromosomal aberrations were detected in 2/18 patients (11 %) before allogeneic SCT, in 12/13 patients (92 %) after 3 months, in all patients after 12 months and in all patients in the retrospective group after allogeneic SCT. The percentage of aberrant cells was significantly higher at all times after allogeneic SCT compared to baseline analysis. Reciprocal translocations were the most common aberrations, but all other types of stable, structural chromosomal aberrations were also observed. Clonal aberrations were observed, but only in three cases they were detected in independently cultured flasks. A tendency to non-random clustering throughout the genome was observed. The percentage of aberrant cells was not different between patients with and without secondary malignancies in this study group.

**Conclusion:**

High-dose chemotherapy and TBI leads to severe chromosomal damage in skin fibroblasts of patients after SCT. Our long-term data suggest that this damage increases with time, possibly due to *in vivo* radiation-induced chromosomal instability.

## Introduction

The most worrisome long-term side effect after successful allogeneic SCT (SCT) is the increased risk of secondary malignancies.

These tumors develop several years after transplantation with rising incidence over time. Besides younger age at transplantation and chronic immunosuppression through chronic graft-versus-host disease (GvHD), intensive conditioning treatment with total body irradiation (TBI) and high-dose chemotherapy has emerged as a prominent risk factor after allogeneic SCT [1–7].

Ionizing radiation has the capacity to induce chromosomal aberrations in a variety of human tissues. Specific chromosomal aberrations in blood cells like losses at chromosomes 5 and 7, inv(16)(p13q22) and aberrations involving 11q23, 17q21, or 21q22 have been identified in chemotherapy-related myelodysplastic syndromes or acute myeloid leukemias after chemotherapy as well as after radioiodine therapy for malignant or benign thyroid disease [8–11]. Less cytogenetic data in secondary solid tumors after radiotherapy are available, showing a diverse spectrum from simple balanced translocations to complex aberrant karyotypes in few cases [12]. Sublethal genomic damage may induce repairing cellular mechanisms after various times of cell cycle arrest or propagation of genetic lesions, which could ultimately lead to malignant transformation [13]. Evidence has accumulated that DNA-damage not only occurs in directly irradiated cells, but also in the progeny of the irradiated cells at delayed times after radiation exposure [13, 14].

Chromosomal aberrations have been described in cultured skin fibroblasts after accidental irradiation in single patients [15, 16] and in a few patients having received radiotherapy [17–19]. No systemic *in vivo* cytogenetic analysis of irradiated skin fibroblasts has been performed in patients.

We investigated the development of cytogenetic aberrations in skin fibroblasts from hematologic patients after conditioning with high-dose chemotherapy and fractionated TBI for allogeneic SCT. A prospective patient cohort was studied before and after allogeneic SCT by taking sequential skin biopsies for chromosomal analysis. Another group of patients, who had received the identical standard high-dose conditioning therapy before allogeneic SCT was studied retrospectively. To our knowledge, this is the first larger prospective as well as retrospective study with a long-term follow-up on *in vivo* induction of cytogenetic aberrations after irradiation.

## Patients and methods

### Patients

Forty-six allogeneic stem cell transplant patients were included in the study. Final analysis comprised 35 of these 46 patients, because 11 of 46 patients were excluded from further analysis for the following reasons: no total body irradiation as part of the conditioning protocol before allogeneic SCT (*n* = 2), equivocal allocation of probes (*n* = 2), insufficient growth of fibroblasts (*n* = 3), metaphases not evaluable after preparation (*n* = 2), early death before 3 months after allogeneic SCT (*n* = 2).

The prospective study group consisted of 18/35 patients. Skin biopsies were taken at three time points: immediately before high-dose conditioning, 3 months and 12 months after allogeneic SCT.

The retrospective study group comprised 17/35 patients. Skin biopsies were obtained once from each patient, in median 38 months after SCT (range 23 to 65 months) (Table 1). The study was approved by the local ethical committee of the Charité Universitätsmedizin Berlin. All patients gave written informed consent.

**Table 1:** Patients’ characteristics in the prospective and the retrospective study group

	Prospective group	Retrospective group
Patients n	18	17
year of transplantation	1999-2001	1995-1999
Age at study entry *median (range)*	37 years (16–49)	40 years (22–59)
Sex		
male	12	8
female	6	9
Diagnosis		
chronic myeloid leukemia	7	9
acute lymphoblastic leukemia	4	4
acute myelogenous leukemia	3	2
myelodysplastic syndrome	2	2
chronic myelomonocytic leukemia	1	0
osteomyelofibrosis	1	0
Donor type	*n* = 17*	
related	9	13
unrelated	8	4
HLA-match		
HLA-identical	15	16
HLA-1-mismatch	2	1
Stem cell source		
bone marrow	3	16
peripheral blood	14	1

Legend to Table 1: *One patient did not proceed to transplantation due to rapidly progressing disease at admission and was therefore only considered for the cytogenetic analysis *before* SCT

### Conditioning regimen

All patients underwent standard high-dose conditioning with fractionated TBI with 6x2 gray (Gy) on three consecutive days and high-dose cyclophosphamide and / or etoposide [20]. Three patients in the prospective group were treated with antithymocyte globulin (ATG, Fresenius, Germany) (3x20 mg/kg) before SCT because of an increased risk of graft failure due to a single HLA-mismatch.

One patient with MDS received additional local radiotherapy to thoracic skin areas and spleen because of biopsy-proven skin infiltrates and enormous splenomegaly.

### GvHD prophylaxis

Patients with a related donor received cyclosporine A (CSA) for approximately 6 months plus short-course methotrexate (MTX) i.v. or prednisone. In patients with unrelated or mismatch donors, GvHD prophylaxis consisted of CSA, MTX and prednisone. Prednisone was tapered off after day 28.

### Processing of skin biopsies

After local anesthesia with lidocaine (1 %), skin samples were obtained as 4 mm punch biopsies. Most biopsies were taken from the upper back. Skin samples were cut into small pieces and placed at the bottom of two culture flasks.

### Fibroblast cultures

Fibroblasts were cultured in fetal calf serum and incubated at 37 °C with 5 % CO_2_-pressure according to standard techniques [21]. Culture medium was changed weekly and cultures were checked regularly for typical fibroblast growth. When sufficient fibroblasts were grown, they were washed, trypsinized and passed to two larger flasks to induce a synchronous cell division. As fibroblasts divide every 16–28 hours in culture, cells were harvested after another 2–3 days, when they changed from a spindle-shaped to a spheroidal form indicating mitosis [22].

### Chromosomal preparation

Colcemide was used to arrest cells in metaphase. After trypsinization, cells were centrifuged and resuspended. Warmed hypotonic solution was slowly added on a shaker and cells were incubated for 20 minutes and then fixed and incubated for another 10 minutes. At least five rounds of fixation, incubation, centrifugation and resuspension were repeated before cells were applied onto an object plate and dried.

### Staining of metaphases

Banding was carried out using Trypsin-Giemsa-banding technique [23]. Plates were incubated for 1 hour at 80 °C. In a first step, object plates were incubated at 20 °C with trypsin and isotonic PBS (phosphate-buffered saline). After rinsing, they were then incubated for 4 minutes in Giemsa staining solution, dried and covered.

### Cytogenetic analysis

For each skin sample, 30 metaphases representing 30 cells *in vitro* were analyzed from the prepared slides. Whenever cells from two separate culture flasks were available, 15 metaphases were analyzed from each flask. Karyotypes were defined according to the International System for Human Cytogenetic Nomenclature [24]. For each skin sample, the percentage of aberrant cells and the mean number of breakpoints per aberrant cell were determined from the 30 selected metaphases. If identical aberrations were detected in two or more metaphases, they were regarded as clonal as defined by others [17, 25]. We separately registered identical aberrations in only one or in both of the flasks set up from one biopsy.

### Statistics

Results of the descriptive analysis are given in median and range.

In order to compare the frequencies of patients with aberrant cells, McNemar-Test was used within the prospective study group.

In order to compare the results of different time points within the prospective study group including the number of aberrant metaphases per 30 metaphases (= the percentage of aberrant cells) and the mean number of breakpoints per aberrant metaphase, a non-parametric factorial analysis for repeated measurements and Wilcoxon rank sum test for paired data were used. To compare different clinical subgroups, Mann–Whitney U Test was performed. Data were analyzed using statistical software (SPSS, version 11.0; SAS, version 8.2). Significance was always assessed at the *p* < 0.05 level, two-sided.

## Results

### Metaphase analysis

In the prospective group, chromosomal aberrations were observed in only two out of 18 patients before SCT. The percentage of aberrant cells was rather low in these two patients and only one clone with a single reciprocal translocation was found, namely t(12;18) in one and t(5;17) in the other patient. One of these patients had received prophylactic cranial irradiation prior to SCT (24 Gy) due to ALL.

Three months after SCT, 12 out of 13 patients had aberrant cells in cytogenetic fibroblast analysis, and 12 months after SCT, all patients displayed cytogenetic aberrations. The number of patients with aberrant cells was significantly higher after SCT at both time points compared to the number of affected patients before SCT (Table 2).

**Table 2:** Cytogenetic fibroblast analysis in the prospective and the retrospective study group

Study group	Prospective					Retrospective
	before SCT (0 mo)	3 months after SCT	p-value (0 vs 3 mo)	12 months after SCT	p-value (0 vs 12 mo)	
pat., n	18	13		11		17
pat. with aberrant metaphases, n (%)	2 (11 %)	12 (92 %)	0.002 (McNemar)	11 (100 %)	0.002 (McNemar)	17 (100 %)
aberrant metaphases/30 metaphases (%)^1^	13 %; 27 %^2^	77 % (0-100 %)	0.002 (Wilcoxon)	63 % (33-97 %)	0.003 (Wilcoxon)	83 % (33-100 %)
mean no. of breakpoints/aberrant metaphase^1^	2.3; 2^2^	3.5 (2.3-6.8)	NA	2.9 (1.6-5.8)	NA	3.8 (1.8-5.4)

Legend to Table 2: pat. = patients. no. = number. ^1^ Results are given in median and range. ^2^ Because only two patients had cytogenetic aberrations before SCT, median and range could not be given and only numbers of these two patients are shown. NA = not applicable because of small number of statistical events

All 17 patients (100 %) in the retrospective group had an aberrant karyotype.

77 % of cells were aberrant three months and 63 % in median 12 months after SCT. Difference between 3 and 12 months after SCT was not significant (*p* = 0.237, Wilcoxon test), but difference between pre-SCT analysis and both time points after SCT was highly significant.

In the retrospective group, a median of 83 % of the analyzed cells was aberrant (Table 2).

The mean number of breakpoints per aberrant cell was too small to allow statistical testing (Table 2).

82 - 94 % of the chromosomal aberrations observed in the prospective and retrospective group were detected in 2 or more cells from one sample and were thus defined as clonal. Most of them were found in only one of the two flasks, however. The frequency of clonal aberrations was not different between the single time points (data not shown). In three patients, we detected cells with identical cytogenetic aberrations in cultures from independently set up pieces of the same biopsy (i.e. in two separately cultured flasks). Karyotype evolution could be observed in 13 patients in our study: in addition to the original aberration(s) defining a clone as such, some cells had acquired further cytogenetic aberrations.

### Types of chromosomal aberrations

**Fig. 1:**
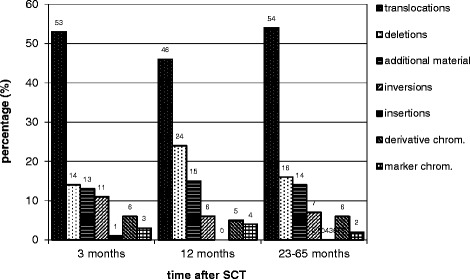
Percentage of different structural chromosomal aberrations in skin fibroblasts. Legend to Fig. 1: chrom. = chromosomes

Only stable, structural chromosomal aberrations were detected. Reciprocal translocations were the most common aberrations, but deletions, additional material of unknown origin, inversions, insertions, derivative and marker chromosomes were also observed. Unstable or numerical aberrations were not seen (Fig. 1). Complex aberrations as defined [26] were identified in five out of 13 patients 3 months after SCT, 2/11 at 12 months after SCT and 8/17 in the retrospective group. Apart from several three-break-translocations, we observed one four-break- translocation (the latter in the retrospective study group).

### Breakpoint analysis

**Fig. 2:**
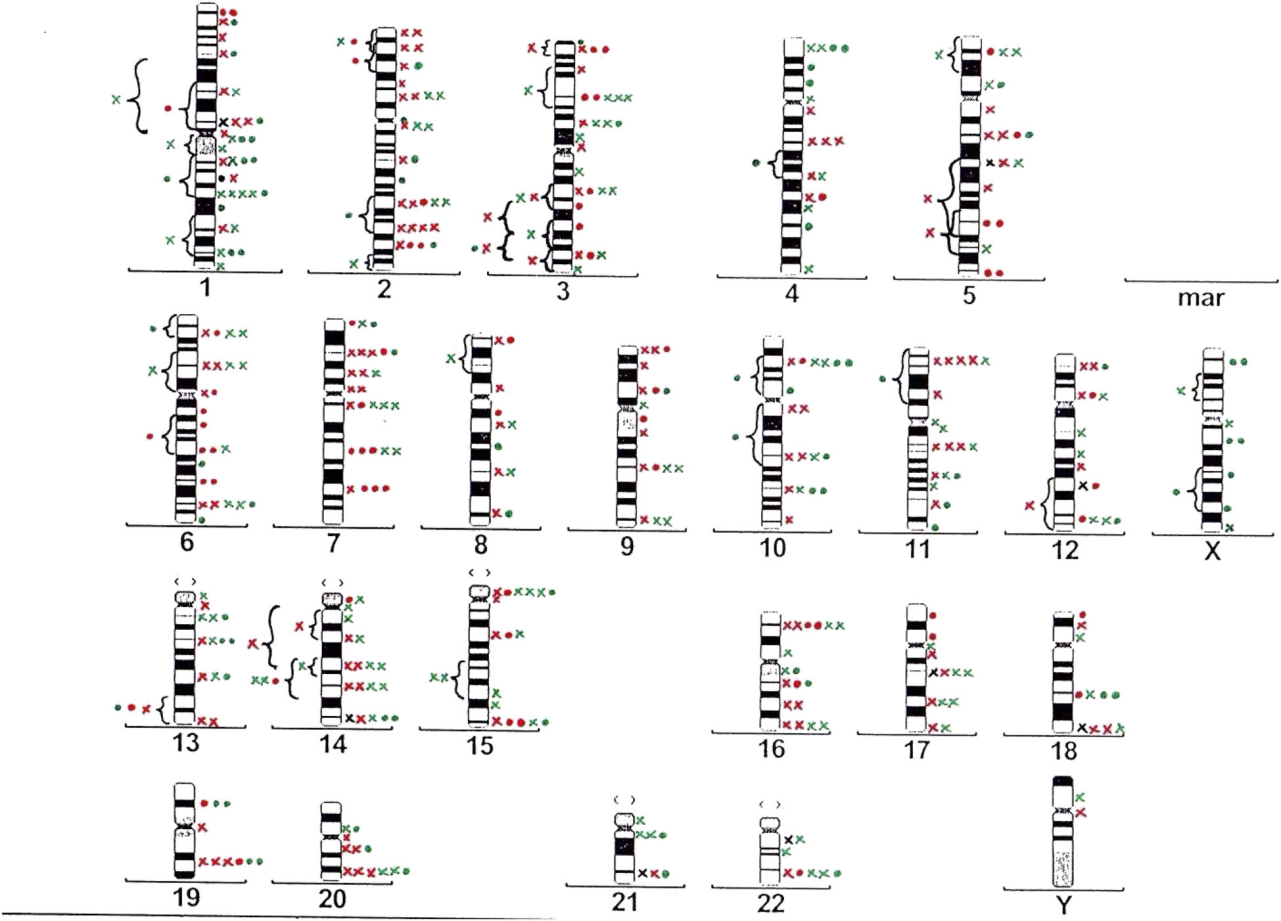
Overview idiogram showing the distribution of all breakpoints in the prospective study group. Legend to Fig. 2: x: breakpoint from a translocation; • breakpoint from an aberration other than a translocation; black: breakpoints before SCT; red: breakpoints 3 months after SCT; green: breakpoints 12 months after SCT

**Fig. 3:**
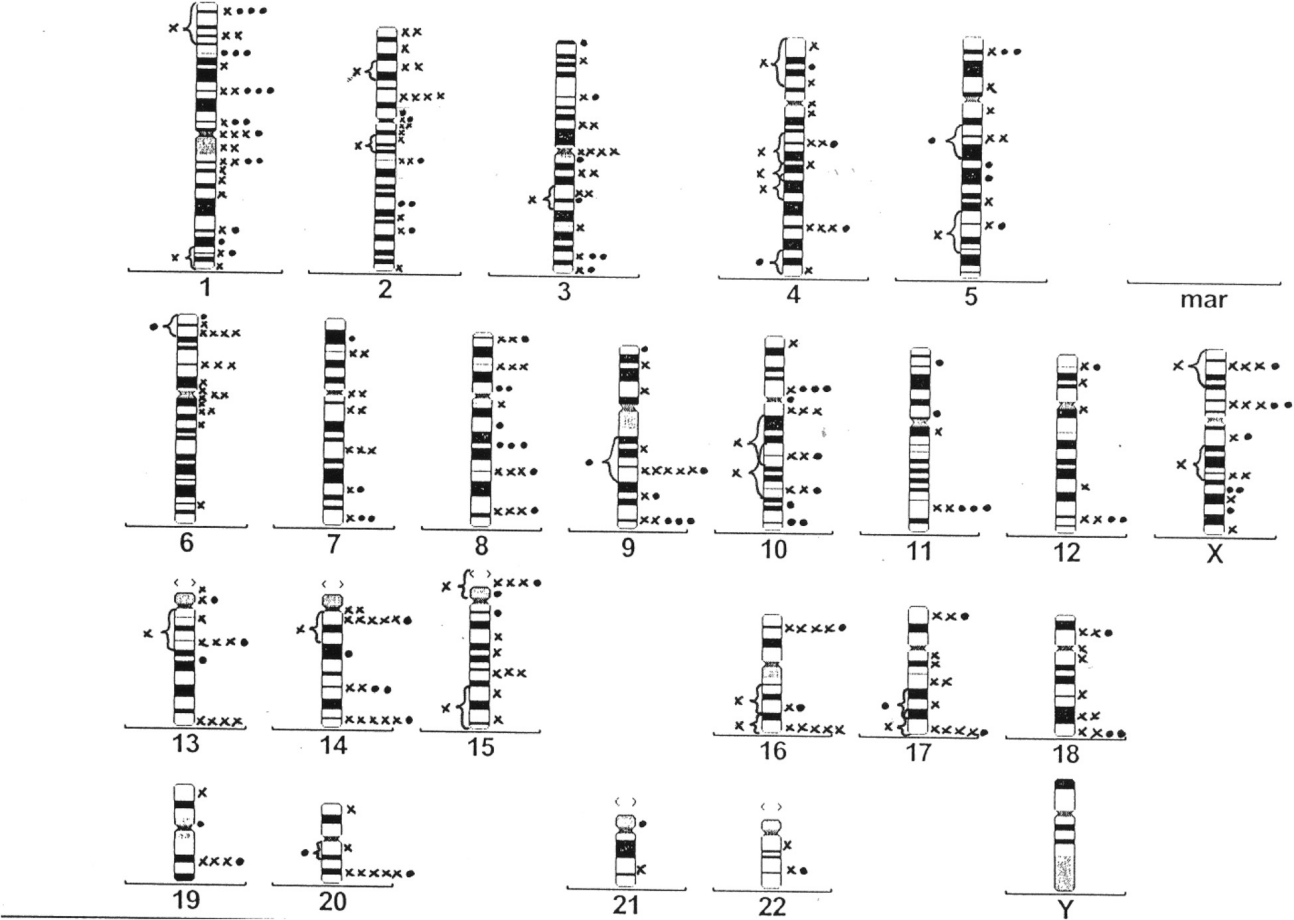
Overview idiogram showing the distribution of all breakpoints in the retrospective study group. Legend to Fig. 3: x: breakpoint from a translocation; • breakpoint from an aberration other than a translocation

Chromosomal breakpoints affected all chromosomes in both groups (except for chromosome Y in the retrospective group) and are illustrated in idiograms (Figs. 2 and 3). Figure 2 shows the breakpoints for the three time points in the prospective group. In Fig. 3, breakpoints of the retrospective group are displayed. Breakpoints observed in clonal aberrations were only counted once for each clone.

There was a tendency to a clustering at a number of chromosome bands. We defined a cluster as a chromosomal band, where at least five breaks were detected (coming from at least 3 different patients) [27]. 15 clusters were detected in the prospective study group and 11 in the retrospective group. In Table 3, the observed and expected percentages of breakpoints for the different chromosomes are shown for the different time points. The expected percentages were calculated assuming that the distribution of breakpoints is proportional to physical length of chromosomes [28].

**Table 3:** Number of different breakpoints and physical length of chromosomes

Chromosome/chromosome arm	Physical length (Mb)	Prospective group			Retrospective group
		before SCT	after 3 months	after 12 months	
1p	128	1	10	5	23
1q	135	1	3	23	16
1	263 (8,0 %)	3	13 (5,8 %)	30 (12,7 %)	39 (10,3 %)
2p	99	-	10	5	12
2q	156	-	12	9	15
2	255 (7,7 %)	-	22 (9,9 %)	14 (5,9 %)	27 (7,1 %)
3p	99	-	9	9	10
3q	115	-	10	9	14
3	214 (6,5 %)	-	19 (8,5 %)	19 (8,1 %)	24 (6,3 %)
4p	56	-	-	7	4
4q	147	-	7	5	15
4	203 (6,2 %)	-	7 (3,1 %)	12 (5,1 %)	21 (5,5 %)
5p	52	-	1	5	4
5q	142	1	12	3	15
5	194 (5,9 %)	1	13 (5,8 %)	9 (3,8 %)	21 (5,5 %)
6p	65	-	6	6	14
6q	118	-	9	6	6
6	183 (5,6 %)	-	15 (6,7 %)	12 (5,1 %)	21 (5,5 %)
7p	65	-	9	4	5
7q	106	-	9	5	10
7	171 (5,2 %)	-	18 (8,1 %)	9 (3,8 %)	15 (4,0 %)
8p	50	-	3	1	8
8q	105	-	4	4	13
8	155 (4,7 %)	-	7 (3,1 %)	5 (3,4 %)	21 (5,5 %)
9p	51	-	6	2	3
9q	94	-	5	4	15
9	145 (4,4 %)	-	11 (4,9 %)	6 (2,6 %)	18 (4.7 %)
10p	44	-	2	6	5
10q	100	-	6	6	15
10	144 (4,4 %)	-	9 (4,0 %)	12 (5,1 %)	22 (5,8 %)
11p	58	-	5	3	2
11q	86	-	5	7	6
11	144 (4,4 %)	-	10 (4,5 %)	10 (4,2 %)	9 (2,4 %)
12p	39	-	4	2	3
12q	104	1	4	5	6
12	143 (4,3 %)	1	8 (3,6 %)	7 (2,9 %)	9 (2,4 %)
13p	16	-	-	1	3
13q	98	-	7	9	11
13	114 (3,5 %)	-	8 (3,6 %)	11 (4,7 %)	14 (3,7 %)
14p	16	-	1	1	-
14q	93	1	9	13	20
14	109 (3,3 %)	1	11 (6,3 %)	14 (5,9 %)	22 (5,8 %)
15p	17	-	2	4	6
15q	89	-	6	7	9
15	106 (3,2 %)	-	9 (6,7 %)	11 (4,7 %)	15 (3,9 %)
16	98 (3,0 %)	-	10 (4,5 %)	9 (3,8 %)	16 (4,2 %)
17	92 (2,8 %)	1	8 (3,6 %)	6 (2,6 %)	16 (4,2 %)
18	85 (2,6 %)	1	5 (2,2 %)	6 (2,6 %)	12 (3,2 %)
19	67 (2,0 %)	-	6 (2,7 %)	4 (1,7 %)	6 (2,4 %)
20	72 (2,2 %)	-	6 (2,7 %)	7 (3,0 %)	10 (2,6 %)
21	50 (1,5 %)	1	5 (2,3 %)	5 (2,1 %)	2 (0,5 %)
22	56 (1,7 %)	1	3 (1,4 %)	5 (2,1 %)	3 (0,8 %)
Xp	62 (1,9 %)	-	-	4	10
Xq	102 (3,1 %)	-	1	6	10
X	164 (5,0 %)	-	2 (0,9 %)	10 (4,3 %)	22 (5,8 %)
Y	59 (1,8 %)	-	1 (0,5 %)	1 (0,4 %)	-
**Sum of breakpoints:**		**10**	**222**	**235**	**379**

Legend to Table 3: Physical length of chromosome arms is given in Mb = Megabases according to [28]. Distribution of breakpoints is shown for chromosome arms and for whole chromosomes. In a few cases (mainly due to derivative chromosomes), breakpoints could only be localized to a chromosome, but not to a certain chromosome arm. Distribution of the breakpoints on the shorter chromosomes 16–22 and Y (chromosome groups E-G) is only shown for the whole chromosome and not for chromosome arms

A higher rate of breakpoints than expected was observed at chromosome arms 1p,1q and 14q (Table 3) [28].

### Clinical parameters and correlation with cytogenetic aberrations

The following clinical parameters were correlated with the percentage of aberrant cells and the mean number of breakpoints detected: patients’ sex, underlying disease, donor type, GvHD of the skin and secondary tumors.

3 months after SCT, the percentage of aberrant cells was significantly higher in patients with acute leukemias compared to patients with other diagnoses (*p* = 0.028). In the retrospective group as well, patients with acute leukemias had a higher mean number of breakpoints per aberrant cell as opposed to patients with other diagnoses (*p* = 0.027, Mann–Whitney U test). All other analyses did not reach statistical significance.

### Secondary malignancies and correlation with cytogenetic aberrations

With an updated follow-up of up to 14 years in the prospective and 18 years in the retrospective group, secondary malignancies affected 7 out of 35 patients, 2 in the prospective and 5 in the retrospective group. Secondary malignancies developed 34 to 158 months (137 months in median) after allogeneic SCT. Two patients had malignant melanoma, another 2 patients had oral squamous cell cancer, 1 patient had uterine cervical cancer, 1 patient had cholangiocellular carcinoma and 1 patient developed bladder cancer. All patients had surgical resection of their secondary malignancies, 1 patient died due to progressive relapse of bladder cancer 3 months after tumor diagnosis, the others survived. Follow-up in the seven patients was 22 months in median after diagnosis of secondary malignancy (3–61 months). Another 4 patients had basal cell cancer of the skin 94 to 120 months after allogeneic SCT, which were successfully removed by surgery.

The percentage of aberrant cells in the retrospective group was not different between the five patients with secondary malignancies (80 % in median, range 67-93 %) and the ten patients without secondary malignancy (83 % in median, range 53-94 %).

## Discussion

We herein describe the time-dependent development of cytogenetic damage induced in skin fibroblasts after conditioning with high-dose chemotherapy and TBI and allogeneic SCT in a larger cohort of patients with a long term follow-up.

The percentage of cells with an aberrant karyotype in our study was significantly higher at all time points after high-dose conditioning compared to the biopsies taken before conditioning. Genetic damage occurred early, because most metaphases from biopsies taken 3 months after SCT already presented an aberrant karyotype.

It is unlikely, that all these aberrations developed *ex vivo* in culture, because most skin metaphases were normal before SCT. In the literature there is only one small series of four pediatric patients after TBI or total lymphoid irradiation, where authors found 49 to 88 % aberrant metaphases in the skin biopsies taken within radiotherapy fields [19]. A few case reports have also been published describing chromosomal aberrations in skin fibroblasts after *in vivo* radiation exposure [15–18, 25], but without comparison to pre-radiation metaphases. To our knowledge, our study is the largest *in vivo* cytogenetic study in irradiated patients with hematologic malignancies, which follows chromosomal aberrations over time.

Patients with acute leukemias, who had received more intensive chemotherapy before TBI had a higher level of cytogenetic damage. Therefore it cannot be excluded that the extent of the observed cytogenetic damage in the present study may have been influenced in part by radiation enhancing effects of chemotherapy. However in a small series of SCT-patients, abnormal karyotypes were only observed in those with TBI, but not in those without [19].

We mainly detected clonal aberrations in our study. Most of them were only seen in single culture flasks, so that *in vitro* clone formation cannot be excluded. A stronger hint to *in vivo* clonality could be found in three of our patients with identical aberrations in cultures from independently set up pieces of the same biopsy. Hints to *in vivo* clone formation come from case reports by others as well [15, 17–19]. The strongest evidence for *in vivo* clone formation was reported by Mouthuy and Dutrillaux, who found cells with identical aberrations in two skin biopsies, which were taken with a time interval of one year [15]. This is a unique finding neither confirmed by us nor others.

A clone is not necessarily completely homogenous since subclones may have evolved exhibiting additional aberrations [24]. This phenomenon described as karyotype evolution was observed in 13 patients of our study.

Reciprocal translocations were the most common type of aberration observed at all time points. In 16 of our patients, complex aberrations were detected. The most complex aberration was a four-break-translocation seen in a patient of the retrospective group.

The high rates of aberrations in our study, karyotype evolution and the presence of complex aberrations are in line with the concept of permanent genomic instability through the cytotoxic effects of irradiation [13, 14, 29].

Studies of *in vitro* irradiated fibroblasts have demonstrated that the radiation-induced breakpoints are distributed non-randomly throughout the genome [27, 30, 31]. In our study some of the clusters were detected at published breakpoints like, for example, 1p22. Both arms of chromosome 1 have been affected frequently in *in vitro* studies of irradiated diploid cells [27]. We are reserved in the interpretation of the results of our breakpoint analysis on the basis of this conventional cytogenetic study. We see hints for a non-random distribution of breakpoints in our study (Figs. 2 and 3, Table 3).

Interestingly, several of the observed clusters were located at bands, where genes for the chromosome instability syndromes have been mapped to. Additionally, a cluster was detected at 14q32, which is known to be a preferential site of chromosomal breakage in ataxia-telangiectasia patients [32]. The genes involved in the chromosome instability syndromes like Ataxia teleangiectasia or Fanconi anemia are critical in the early detection of induced damage and subsequent induction of cellular response to repair the damage [13, 33]. Patients with these or other related disorders have a dramatically increased malignancy risk [33]. An interesting task for future studies would be to compare known gene loci and breakpoints in these cancer-prone disorders with radiation-induced aberrations with molecular tools. We hypothesize, that a congruence of breakpoints induced by radiation with chromosomal breakage sites in chromosome instability syndromes would support the concept of radiation-induced instability through disturbances in DNA repair as suggested by others [13].

Due to the limited number of patients in our experimental study, the rates of secondary malignancies cannot be compared with large register data [1–7], although the 20 % cumulative incidence of secondary malignancies seems rather high. As virtually all patients had cytogenetic aberrations in skin, additional cellular mechanisms must be involved in carcinogenesis in these patients. It also seems very likely, that organ-specific cellular changes are involved in organ carcinogenesis.

More advanced technologies like FISH, SKY and molecular techniques have evolved rapidly with more data being available on mechanisms of cell damage and repair due to radiation injury [34–36]. These data, however, are either from cell culture systems and mice models or did not involve patients with TBI, while our data were from *in vivo* irradiated patients’ fibroblasts. Experimental approaches and organ-specific analyses might detect specific cytogenetic and molecular aberrations, which would increase our understanding of organ-specific radiation-induced cellular injury and carcinogenesis.
